# Adherence to Dietary Guidelines in Adults by Diabetes Status: Results From the 2012 Mexican National Health and Nutrition Survey

**DOI:** 10.3390/nu12113464

**Published:** 2020-11-12

**Authors:** Nancy López-Olmedo, Satya Jonnalagadda, Ana Basto-Abreu, Alan Reyes-García, Carolyn J. Alish, Teresa Shamah-Levy, Tonatiuh Barrientos-Gutierrez

**Affiliations:** 1 Center for Population Health Research, National Institute of Public Health, Cuernavaca, Morelos 62100, Mexico; nancy.lopez@insp.mx (N.L.-O.); ana.basto@insp.mx (A.B.-A.); rega.nutri@gmail.com (A.R.-G.); 2 Abbott Nutrition, Columbus, OH 43219, USA; satya.jonnalagadda@abbott.com (S.J.); calish@columbus.rr.com (C.J.A.); 3 Evaluation and Surveys Research Center, National Institute of Public Health, Cuernavaca, Morelos 62100, Mexico; tshamah@insp.mx

**Keywords:** dietary patterns, diabetes, Mexican adults

## Abstract

The aims of the present study were to compare the adherence to dietary guidelines and evaluate potential differences in nutrient profiles among adults by diabetes status. We used the Mexican Alternate Healthy Eating Index (MxAHEI) to evaluate adherence to dietary guidelines. We calculated the MxAHEI scores (total and by dietary component) with scales from 0 (non-adherence) to 100 (perfect adherence) based on a food frequency questionnaire. Mean daily intakes of macronutrients and micronutrients (g, mg, mcg/1000 kcal per day) were also estimated by diabetes status. Sex-specific, multivariable linear regression models were estimated to test whether MxAHEI scores as well as nutrient intakes were different by diabetes status. Mexican adults had low adherence to the dietary guidelines irrespective of their diabetes status (score < 50 points). Among men, the MxAHEI score was 2.6 points higher among those with diabetes than those without diabetes (46.9; 95% confidence intervals (CI): 44.6, 49.2 vs. 44.3; 95% CI: 44.2, 45.6, respectively). Among women, the total MxAHEI score was similar in individuals with diabetes compared to those without diabetes. Lower intakes of carbohydrates and added sugars and higher intakes of protein, calcium, and zinc were observed in individuals with diabetes. Our findings support the development of strategies focused on promoting dietary patterns that can help to prevent and control the disease.

## 1. Introduction

In 2016, type 2 diabetes was declared an epidemiological emergency in Mexico. Diabetes is the second cause of death in the country, where it accounts for 15.2% of all deaths annually and is the leading cause for years of life lost [1]. The prevalence of diagnosed diabetes in Mexico is 9.4% of all adults, representing 6.5 million people with diabetes. Complications of diabetes are frequent, 21.1% of people with type 2 diabetes mellitus experience microvascular complications (diabetic foot, retinopathy, or nephropathy), and 3.4% have macrovascular complications [2]. In 2011, diabetes costs in Mexico were estimated to be 7.7 billion US dollars each year [2], of which 44% were direct healthcare costs [3].

Nutrition therapy is a cornerstone of the management and prevention of diabetes. Recent updates to the American Diabetes Association (ADA) nutrition guidelines states that treatment and prevention of the disease should be focused on the overall diet more than a single nutrient or food group [4]. In individuals with diabetes, higher adherence to healthy dietary patterns have been associated with lower glycated hemoglobin levels and lower risk of diabetes complications [3]. However, adherence to healthy diets among people with diabetes can be challenging and remains a fundamental barrier to diabetes management worldwide [5,6].

Dietary indices have been recommended to evaluate adherence to dietary recommendations among individuals or populations. The Alternate Healthy Eating Index (AHEI) represents a measure of diet quality in relation to nutrition guidelines to manage and prevent chronic diseases such as diabetes because it was developed on evidence-based recommendations that specific foods and nutrients can have synergistic or antagonistic effects in the development or management of chronic diseases, particularly cardiometabolic diseases [2,7,8,9].

In Mexico, 22% of the population with diabetes reported modifying their diets as part of their diabetes management [2]. However, no study has evaluated the diet quality of adults with diabetes or examined whether diet quality differs based on diabetes status at the national level in Mexico. We aimed to compare the diet quality between adults with and without self-reported diabetes using food frequency data from the 2012 Mexican National Health and Nutrition Survey (ENSANUT), and the AHEI adapted to the Mexican context [10]. We also evaluated the intake of macronutrients and micronutrients by diabetes status as a secondary aim. Conducting this type of analysis is useful to understand the dietary behaviors of people with diabetes and could provide a platform to develop food and nutrition policies for this population.

## 2. Materials and Methods

### 2.1. Data Source and Population

The 2012 ENSANUT is a cross-sectional, multistage, stratified, and cluster-sampled survey representative of urban and rural areas, at the national, regional, and state levels in Mexico. The design and methods are described elsewhere [11]. Briefly, the 2012 ENSANUT was conducted between October 2011 and May 2012 and obtained information about sociodemographic, nutrition, and health characteristics from 96,031 people. Dietary information was obtained using a validated Semi-Quantitative Food Frequency Questionnaire (SFFQ) [12,13] applied to a random subsample of 11% of the sample. This study was conducted according to the guidelines in the Declaration of Helsinki, and all procedures involving human participants were approved by the Ethics Committee of the Mexican National Institute of Public Health (project number 1033, approval 1108). Written informed consent was obtained from all participants under study.

We included males and non-pregnant and non-lactating females >18 years (*n* = 7 512) and excluded individuals with incomplete health, anthropometric, or sociodemographic information (*n* = 400). We also excluded 56 individuals with extreme total energy intake (expressed as the ratio of total energy intake to estimated energy requirement in logarithmic scale +/−3 standard deviations (SD)), as previously described [14]. The analytical sample was composed of 2762 participants, who were stratified by sex and diabetes status.

### 2.2. Dietary Information

Trained interviewers collected dietary information using the 140-item Semi-Quantitative Food Frequency Questionnaire (SFFQ) adapted by the Mexican National Institute of Public Health. For each food, participants reported the frequency and the number of standard portions consumed over seven days prior to the interview. We then converted the reported intake into grams or milliliters per day. We considered edible portion and density factor to obtain net grams of solid food consumed and milliliters consumed from beverages, respectively. It is relevant to notice that the SFFQ has individual foods and mixtures (dishes with sets of default ingredients). For the present analysis, we disaggregated these mixtures into their component ingredients using standard recipes. Energy and nutrient values were estimated using a Food Composition Table compiled by the Mexican National Institute of Public Health in Morelos, Mexico [15]. Added sugar values were estimated with methodology used by Sánchez-Pimienta et al. [16]. The portion sizes of each SFFQ item were estimated based on the Mexican Equivalent Food System [17].

### 2.3. Mexican Healthy Eating Index (MxAHEI)

We evaluated the adherence to dietary guidelines using the Mexican Alternate Healthy Eating Index (MxAHEI) [10], which is based on the original AHEI-2010 components and criteria for maximum and minimum scores [8,18]. Briefly, we identified portion intakes of 12 components: seven for which higher intakes are recommended (vegetables, whole fruit, fiber from cereals, legumes, nuts, and long chain omega-3 (n-3) fatty acids (eicosapentaenoic acid—EPA and docosahexaenoic acid—DHA), and polyunsaturated fats), and five that must be limited or avoided (sugar-sweetened beverages (SSBs), red and processed meats, sodium, trans fats, and alcohol). Each component was scored on a 0 to 5- or 9-point scale. The component scores were summed to obtain the total MxAHEI score, which can range from 0 (non-adherence) to 100 (perfect adherence). Details of the index components and the score standards are summarized in Table 1.

### 2.4. Health and Anthropometric Information

We determined self-reported diabetes status (yes/no) from responses to the question “Have you ever been told by a physician that you have diabetes or high sugar in your blood? Self-reported hypertension status was defined from responses to the question “Have you ever been told by a physician that you have high blood pressure or hypertension? Smoking status was self-reported and categorized as “current”, for study participants who reported smoking at least 100 cigarettes during their lifetime and having smoked during the last 30 days; “former”, for those who reported smoking at least 100 cigarettes during their lifetime and who did not currently smoke; and “never” for those who never smoked. We calculated the Metabolic Equivalents (MET) per week for each individual based on reported measures of minutes per week of walking, and moderate and vigorous physical activities. Trained personnel obtained anthropometric measurements using standard procedures [19]. Body weight was measured with participants wearing light clothing using digital scales (Seca © 872 digital floor scale, Gmbh & Co., Hamburg, Germany); stadiometers (Dyna-top ©, model E-1, Multimed, Monterrey, Mexico) were used to measure height. We calculated the body mass index (BMI) using the standard equation and categorized the results into normal, overweight, and obese based on World Health Organization classification [20].

### 2.5. Sociodemographic Variables

We defined rural areas as locations with < 2500 inhabitants and urban areas as locations with ≥ 2500 inhabitants, and we defined regions as North, Center, and South. A wealth index was constructed using principal components analysis that was applied to household characteristics and assets [21]. The index was then classified into three categories (low, medium, and high) using tertiles of the distribution of index as cut-off points. Education level was categorized as elementary, middle, high school, and college or more, according to the last grade of studies completed.

### 2.6. Statistical Analysis

We conducted a descriptive analysis by sex and diabetes status using means or percentages and 95% confidence intervals (CI). To test whether the MxAHEI diet scores (total and by dietary component) were different between individuals with and without diabetes, we performed a sex-specific linear regression models unadjusted and adjusted for age, hypertension status, BMI, smoking status, educational level, wealth index, rural/urban area and geographic region. We also estimated daily intakes of macronutrients, fiber and micronutrients (g, mg, or mcg per 1000 kcal per day). Similarly, we compared intakes of nutrients between adults with and without diabetes using sex-specific linear regression models unadjusted and adjusted for the same variables used to analyze the MxAHEI diet score.

Mean values and 95% confidence intervals were used to present the average dietary scores and nutrient intakes for men and women with and without diabetes. We used population weighted factors for all the statistical analyses and considered the survey’s complex sampling design. Statistical tests were two-tailed and considered significant at *P* < 0.05. All analyses were carried out using Stata version 13 (StataCorp, Stata Statistical Software, Release 13, 2013. College Station, Texas, United Stated of America: StataCorp LP) [22].

## 3. Results

Participant characteristics are presented in Table 2. The 2762 individuals analyzed represent 62.1 million adults, of whom 41.3% were men; 7.2% of men and 9.8% of women reported being diagnosed with diabetes. Compared to participants without diabetes, participants with diabetes were older, less educated, and had lower physical activity. They also had a higher prevalence of hypertension and being overweight and had lower energy intake. In addition, among adults with diabetes, the time since diagnosis was longer among women than men. Moreover, regardless of the diabetes status, the mean of total MxAHEI score was less than half of the maximum score (100).

Among men, using the unadjusted models, the score of total MxAHEI and whole fruit and SSBs components were higher in those with diabetes than those without diabetes (Table S1). Similar results were observed in adjusted models (Table 3). The adherence to the total MxAHEI was higher in individuals with diabetes than those without diabetes (46.9; 95% CI: 44.6, 49.2 vs. 44.3; 95% CI: 44.2, 45.6, respectively). Specifically, participants who had diabetes had higher intakes of fruits than those without diabetes. Also, those with diabetes had lower intakes for SSBs (higher MxAHEI score) than men without diabetes. The intakes of the remaining components were similar between those with diabetes and those without diabetes. However, the intakes of most dietary components were slightly higher (higher MxAHEI score) among individuals with diabetes compared to those without diabetes. Inversely, the dietary components where the recommendation is to limit intake showed slightly lower intakes (higher MxAHEI score) among men with diabetes than those without diabetes, except for red and processed meats and sodium, where the intake observed was in the opposite direction.

Among women, the total MxAHEI score was similar in individuals with diabetes compared to those without diabetes (45.3; 95% CI: 43.9, 46.7 vs. 45.1; 95% CI: 44.5, 45.7, respectively). There were significant differences in omega-3 and SSBs scores. Women with diabetes had lower intakes for omega-3 (lower MxAHEI score); and SSBs (higher MxAHEI score) compared to those without diabetes in both, unadjusted and adjusted models (Tables S1 and S2, respectively). The intakes of most dietary components were similar across diabetes categories. Nevertheless, the intakes of most dietary healthy components were lower (lower MxAHEI score) among individuals with diabetes than among those who did not have diabetes.

Table S3 and Table 4 present sex-specific unadjusted and adjusted proportional macronutrient intakes by diabetes status. Among men with diabetes, we observed higher intakes of protein and total, monounsaturated and saturated fatty acids, and lower intakes of carbohydrates and added sugars compared to men without diabetes. Although not significant, the intakes of the remaining nutrients were higher among individuals with diabetes versus those without diabetes. The intake of total and monounsaturated fatty acids was not statistically different between men with and without diabetes in adjusted models.

Women with diabetes had lower intakes of total and added sugars than women without diabetes. On the other hand, total protein intake was higher in women with diabetes than women without diabetes. While not significant, the intakes of carbohydrates, monounsaturated, and polyunsaturated fatty acids were lower in women with diabetes than those without diabetes. Conversely, the intakes of fiber, total and saturated fatty acids, and omega-3 were higher among women with diabetes versus those without diabetes. The difference in total protein intake between women with and without diabetes was no longer statistically significant in the adjusted models.

Table S3 and Table 5 show sex-specific unadjusted and adjusted proportional mean daily micronutrient intakes by diabetes status. Men with diabetes had higher daily intakes of vitamins A, D, B6, and B12 and folate, calcium, magnesium, zinc, and potassium than did men who did not have diabetes. While not significant, similar trends were observed for intakes of the remaining micronutrients. In the adjusted model, the difference in the intakes of vitamins A, D, B6, and B12 between men with and without diabetes was no longer significant.

Among women, the average intakes of calcium, magnesium, zinc, and potassium were higher among participants who had diabetes than among those who did not have diabetes. For the remaining micronutrients, although the results were not significant, the intakes were higher among women with diabetes versus women without diabetes, except for vitamin E, C, and sodium. The potassium intake was not different between women with and without diabetes in adjusted models.

## 4. Discussion

We compared the diet quality of self-reported declared Mexican adults with and without diabetes. Overall, the adherence to the MxAHEI was poor among Mexican adults, with a score below 50 out of 100, regardless of the diabetes status. We found a higher adherence to the MxAHEI among men with diabetes than among men who did not have diabetes (score of 46.9 vs. 44.3, respectively). Specifically, adherence to the recommended intakes for fruits and SSBs was greater among men with diabetes versus those without diabetes. Among women, the total MxAHEI score was similar in individuals with diabetes compared to those without diabetes. Specifically, women with diabetes had lower intakes of omega-3 and SSBs than did those who did not have diabetes. We also found higher intakes of protein, saturated fatty acids, folate, calcium, magnesium, zinc, and potassium, and lower intakes of carbohydrates and added sugars among men with diabetes compared to those without diabetes. Finally, among women, the intakes of total and added sugars were lower, while calcium and magnesium were higher among individuals with diabetes versus those without diabetes.

In our study, we found that overall adherence to the MxAHEI was poor in both men and women, a finding consistent with previous studies that reported low adherence to dietary recommendations in the general population, characterized by low consumption of fruits and vegetables and high consumption of meat [23,24,25]. In addition, our results are in line with the findings of a cross-sectional study conducted in Spain, where it was found that adherence to the AHEI in adults was low, regardless of diabetes status [26].

The difference in the total MxAHEI score among men by diabetes status can be attributed to different intakes of some dietary components, particularly a higher intake of fruits and a lower intake of SSBs among individuals with diabetes than among those without diabetes. Similar to men, the consumption of SSBs was significantly lower among women with diabetes compared to their counterparts who did not have the disease. However, adherence to recommended consumption was slightly lower in women with diabetes than women who did not have diabetes in most dietary components, particularly in omega-3 fatty acids, which may explain our finding that there was no significant differences in the total MxAHEI among women who had diabetes and those who did not.

These findings are consistent with other population-based studies that analyzed dietary behaviors by diabetes status. Fitzgerald et al. and Nöthlings et al., found lower intakes in SSBs and slight increases in intakes of food groups considered healthy among individuals with diabetes than in those without diabetes [27,28]. The better adherence to recommendations of fruits and SSBs among individuals with diabetes was an expected result in our study. As part of medical nutrition therapy, individuals with diabetes are encouraged to increase their consumption of fruits, vegetables, and legumes while reducing the consumption of foods containing refined carbohydrates and saturated fats [29]. In our study, both men and women with diabetes had a better score for SSBs, however, their consumption was far below the perfect adherence (zero consumption of SSBs). The SSBs score in men and women was 3.9 and 5.7, respectively, from a maximum of 9.

We also observed that the overall adherence to dietary recommendations was higher in men with diabetes versus women with diabetes. Despite changes in gender roles over the last decades in Mexican society, a considerable gender gap remains in the time spent on unpaid work, such as household chores (6.4 h for Mexican women, 2.3 h for Mexican men) [30]. It has also been reported that women, rather than men, spend more time as caregivers. [31]. We can postulate that women who spend more time engaged in unpaid work and in caregiver roles may perceive they have limited ability to engage in self-care behaviors to manage their diabetes or other chronic diseases. These gender inequalities might decrease the ability for women to take care of their own, and this may hamper their lifestyle changes, including dietary behaviors, when diagnosed with diseases. Also, it has been observed that women may not be willing to change their lifestyles to not disrupt the family [32]. Future studies should examine dietary change dynamics after chronic disease diagnosis with a gender perspective, as social roles could play an important role in dietary modifications. For instance, studies could compare the similarity of diets in members of the same household, to assess if men have more dissimilar diets when diagnosed with diabetes than women under the same disease conditions.

Our findings of reduced intakes of carbohydrates and added sugars among adults with diabetes are consistent with previous studies in adults from several European countries and among women in the United States [33,34,35]. These results are expected given the link between dietary sugar and serum glucose or “blood sugar” levels [36]. Reducing the consumption of foods with a high glycemic index is a key nutritional recommendation of many international diabetes associations [37,38], including those in Mexico [39]. Part of this recommendation involves evenly distributing carbohydrate intake across meals and snacks throughout the day. Because carbohydrates have the greatest impact on blood glucose levels in diabetes, individuals may opt to limit foods containing carbohydrates as a way to manage their diabetes. In our study, the findings for added sugar and SSBs were in the same direction, which was foreseeable given that SSBs are the greatest contributor to added sugar intake in Mexico [16].

We observed that protein intake was higher in men and women with diabetes than those who did not have diabetes, in line with findings among adults from European countries [33,34,35]. However, we also found that the intake of total fat, and specifically the intakes of saturated and monounsaturated fatty acids, were higher among men with diabetes than in men without diabetes. Only the study by Virtanen et al. found a higher intake of total fatty acids among men with diabetes from Finland and the Netherlands [33]. Given that the reduction of one macronutrient is substituted with others [40], we hypothesize that among individuals in our study, the reduction in carbohydrates was replaced by additional proteins and fatty acids.

We observed differences in select micronutrients based on diabetes status. In general, the intakes of calcium, magnesium and zinc were higher in adults with diabetes than in those without diabetes. However, the trend in these micronutrients are not generalized to the consumption of the components of the MxAHEI, particularly in vegetables, legumes, nuts, and red meat. The inconsistency in findings between nutrient intakes and dietary components can be partly explained by the truncated nature of the index score. In other words, the probable variability in food intake among individuals with the lowest or highest scores was no longer considered when the score is assigned. Furthermore, the MxAHEI does not consider dairy, which contributes to the intake of several micronutrients, particularly calcium. We also observed that potassium and folate intakes were higher among men with diabetes than in men without diabetes. This is similar to a previous study carried out in European populations with and without diabetes, where similar trends were observed [33]. These results are linked with the higher consumption of fruits among men with diabetes because dietary potassium and folate can be found in a wide variety of fruits [41]. Lower intakes of folate and potassium among individuals with diabetes, particularly among women, raises the issue of improving intakes in this population in view that these micronutrients play a significant role in insulin synthesis and sensitivity [42,43,44].

Our analysis has several limitations that are important to acknowledge. First, even though the SFFQ provides a good estimation of usual intake and therefore of diet quality, it is not the optimal instrument for estimating absolute nutrient intakes. We addressed this limitation by reporting proportional intakes. Second, there is a potential loss of accuracy to detect differences between micronutrient intake and MxAHEI components due to the truncated nature of the latter. However, the MxAHEI is an adapted version of the AHEI-2010, which is the only diet quality index validated against different chronic diseases, including diabetes [8,18,45,46,47]. Specifically, the MxAHEI and AHEI-2010, unlike other dietary indices, include the SSBs component, which largely accounted for the differences found by diabetes status, as also observed in a previous study [9]. Third, we cannot rule out the possibility of social desirability bias [48], which may impact the reported dietary intake data of adults who have diabetes, relative to those who do not have diabetes. People who have diabetes are more likely to receive dietary counseling and education about diet than are individuals who do not have diabetes. Fourth, participants were not asked to respond whether they were diagnosed with type 1 or type 2 diabetes. However, since type 1 diabetes is usually diagnosed in children and adolescents [49] and is a less common type of diabetes [50], a low proportion of Mexican adults likely have type 1 diabetes. In our representative sample, 2.7% was diagnosed with diabetes during childhood or adolescence. Therefore, it is more likely that our results characterize adults with type 2 diabetes. Moreover, diet recommendations are similar between people with type 1 and type 2 diabetes [49]. Fifth, the duration or time since diagnosis is another factor that can affect the adherence to dietary recommendations. However, we could not conduct analyses among men and women with diabetes by time since diagnosis because the sample size is limited. Future studies with larger samples will help to understand the role of time since diagnosis on dietary recommendations adherence. Finally, we did not include physical activity in the regression models due to the poor validity of the International Physical Activity Questionnaire short form for assessing physical activity among Mexican adults [51]. Despite these limitations, the results presented provide an overview of the adherence to dietary guidelines in adults and how this adherence may be different by diabetes status in a representative sample of Mexican men and women. This offers information to understand the disparities in diet and health in Mexico.

## 5. Conclusions

In conclusion, we observed higher adherence to dietary recommendations among Mexican adults with diabetes than adults who did not have diabetes, especially among men. These findings suggest that Mexican adults with diabetes are adhering to the dietary guidelines in some extent. However, we emphasize that MxAHEI scores, total and by component, were well below the recommendations. Therefore, it is necessary to reinforce the implementation and evaluation of programs that promote healthy dietary patterns in the Mexican population. It is also important to consider in such strategies the potential gender inequalities for reaching the dietary recommendations.

## Figures and Tables

**Table 1:** Mexican Alternate Healthy Eating Index. Components and scoring criteria.

Food Component		MxAHEI	
	Maximum Score	Criteria for Minimum Score (0)	Criteria for Maximum Score
Higher Intake Recommended			
Vegetables	9	0 servings	≥5 servings
Whole fruit	9	0 servings	≥4 servings
Fiber from cereals	9	0 g	≥15 g
Legumes	5	0 servings	≥1 serving
Nuts	5	0 servings	≥1 serving
Long-chain (n-3) fatty acids (EPA + DHA)	9	0 mg	≥250 mg
Polyunsaturated fats ^a^	9	≤2% of total energy intake	≥10% of total energy intake
**Limited Intake Recommended**			
SSBs	9	≥1 serving	0 servings
Red and processed meat	9	≥1.5 serving	0 servings
Sodium	9	>2 g	≤1.5 g
Trans fats	9	≥4% of total energy intake	≤0.5% of total energy intake
Alcohol			
Women	9	≥2.5 drinks	0.5–1.5 drinks
Men		≥3.5 drinks	0.5–2.0 drinks
Total score	100		

^a^ Excluding long-chain (n-3) fatty acids. MxAHEI, Mexican Alternate Healthy Eating Index; DHA, docosahexaenoic acid; EPA, eicosapentaenoic acid; SSBs, sugar-sweetened beverages.

**Table 2:** Sex-stratified characteristics of Mexican adults by diabetes status (*n* = 2762) ^a^.

Variables	Men				Women			
	Diabetes		Non-Diabetes		Diabetes		Non-Diabetes	
*n*	82		1060		159		1461	
N (millions)	2.5		26.3		3.7		29.5	
	Mean	SE	Mean	SE	Mean	SE	Mean	SE
Age (years)	61.3	3.9	41.8	0.7	56.9	1.9	41.1	0.6
Time since diagnosis (years)	8.9	0.9			11.1	1.4		
BMI (kg/m^2^)	28.1	0.8	27.3	0.2	29.2	0.7	29.1	0.3
Total energy intake (kcal/day)	1936	92.7	2216	41.7	1695	82.7	1817	33.6
Total MxAHEI	48.0	1.2	44.4	0.3	46.6	0.6	45.1	0.4
Physical Activity (MET-min/week)	3273.4	855.8	7048.5	432.1	2847.7	635.4	4422.1	324.6
	%	SE	%	SE	%	SE	%	SE
Hypertension	53.6	9.0	9.5	1.4	45.0	5.6	17.1	1.5
BMI Categories								
Normal	23.0	7.0	37.0	2.3	23.5	5.8	27.8	1.8
Overweight	50.0	9.2	35.5	2.2	38.1	5.4	33.0	1.7
Obesity	27.0	6.9	28.0	2.2	38.4	5.5	39.2	1.9
Area								
Urban	83.6	4.3	75.4	1.8	85.4	3.5	75.0	1.5
Rural	16.4	4.3	24.6	1.8	14.6	3.5	25.0	1.5
Region								
North	16.2	4.4	19.8	1.5	23.4	4.3	19.4	1.3
Central	61.8	7.7	47.9	2.5	44.4	6.8	48.9	2.0
South	22.0	6.1	32.3	2.1	32.2	5.3	31.7	1.8
Tertiles of Wealth Index								
Low	17.3	5.9	27.3	1.9	29.6	5.5	25.2	1.6
Medium	17.5	4.6	31.5	2.1	31.1	4.7	33.2	2.2
High	65.2	7.6	41.2	2.1	39.3	6.4	41.6	2.2
Education level								
Elementary School or less	61.4	8.5	41.3	2.3	81.4	4.0	42.6	2.1
Middle School	13.1	4.5	32.5	2.7	12.3	3.1	25.9	1.7
High School	11.5	4.9	14.0	1.6	3.8	2.1	18.1	1.4
College or more	14.0	6.4	12.2	1.5	2.5	1.3	13.4	1.7
Smoking status								
Never	35.2	10.0	45.8	2.4	74.9	5.8	81.0	1.7
Former	39.1	10.7	28.0	2.6	12.6	3.2	10.0	1.3
Current	25.7	6.9	26.2	2.6	12.5	5.1	9.0	1.2

^a^ Values are presented as means or percentages and SEs. Estimates were weighted to adjust for unequal probability of sampling and to be nationally representative. MET—Metabolic Equivalents; BMI—Body Mass Index. SE—standard error; MxAHEI—Mexican Alternate Healthy Eating Index.

**Table 3:** Sex-stratified adjusted Mexican Alternate Healthy Eating Index Score and components in Mexican adults by diabetes status (*n* = 2762) ^a^.

Variables		Men						Women					
		Diabetes		Non-Diabetes				Diabetes		Non-Diabetes			
*N*		82		1060				159		1461			
	Maximum Points	Mean	95% CI	Mean	95% CI	Diff Diab vs Non-Diab	*p*-Value	Mean	95% CI	Mean	95% CI	Diff Diab vs. Non-Diab	*p*-Value
Total Scores	100	46.9	44.6, 49.2	44.3	44.2, 45.6	2.6	0.018	45.3	43.9, 46.7	45.1	44.5, 45.7	0.2	0.807
Component Scores													
Higher intake recommended													
Vegetables	9	6.6	5.6, 7.5	6.0	5.7, 6.3	0.6	0.318	5.9	5.3, 6.5	6.3	6.0, 6.5	−0.4	0.245
Whole fruit	9	3.9	3.1, 4.6	2.9	2.7, 3.2	1.0	0.015	2.9	2.4, 3.3	3.3	3.1, 3.5	−0.4	0.081
Fiber from cereals	9	2.2	1.7, 2.8	2.2	2.0, 2.3	0.0	0.763	1.9	1.5, 2.3	2.1	1.9, 2.2	−0.2	0.335
Nuts	5	0.3	0.1, 0.5	0.3	0.2, 0.3	0.0	0.684	0.2	0.1, 0.2	0.2	0.1, 0.2	0.0	0.780
Legumes	5	3.6	3.1, 4.1	3.5	3.3, 3.7	0.1	0.696	3.0	2.5, 3.5	3.1	2.9, 3.3	−0.1	0.761
EPA + DHA	9	3.2	2.5, 3.9	2.9	2.7, 3.1	0.3	0.347	1.8	1.4, 2.2	2.6	2.4, 2.7	−0.8	0.001
Polyunsaturated Fats	9	6.0	5.3, 6.6	5.7	5.5, 5.9	0.3	0.443	5.2	4.7, 5.8	5.7	5.5, 5.9	−0.5	0.138
Limited intake recommended													
SSBs	9	3.9	2.6, 5.1	2.9	2.2, 3.8	1.0	0.010	5.7	4.9, 6.5	4.3	4.0, 4.6	1.4	0.001
Red/processed meat	9	3.9	3.2, 4.7	4.1	3.8, 4.4	−0.2	0.758	4.8	4.1, 5.5	4.8	4.5, 5.1	0.0	0.962
Sodium	9	2.7	1.6, 3.8	3.4	3.0, 3.8	−0.7	0.245	4.9	3.9, 5.9	3.9	3.6, 4.3	1.0	0.056
Trans Fats	9	9.0	9.0, 9.0	9.0	9.0, 9.0	0.0	0.471	9.0	9.0, 9.0	9.0	9.0, 9.0	0.0	0.995
Alcohol	9	1.60	0.8, 2.5	1.44	1.2, 1.7	0.2	0.713	0.04	−0.04, 0.12	0.01	0.001, 0.03	0.03	0.557

^a^ Sex-specific multivariable linear regression models were used to predict dietary scores according to diabetes status and adjusting for age, hypertension status, body mass index, smoking status, educational level, wealth index, rural/urban area, and geographic region. Estimates were weighted to adjust for unequal probability of sampling and to be nationally representative. CI—confidence intervals; DHA—docosahexaenoic acid; EPA—eicosapentaenoic acid—SSBs, sugar-sweetened beverages.

**Table 4:** Sex-stratified adjusted intakes of macronutrients in Mexican adults by diabetes status (*n =* 2762) ^a^.

Variables	Men						Women					
	Diabetes		Non-Diabetes				Diabetes		Non-Diabetes			
*N*	82		1060				159		1461			
	Mean	95% CI	Mean	95% CI	Diff Diab vs Non-Diab	*p*-Value	Mean	95% CI	Mean	95% CI	Diff Diab vs. Non-Diab	*p*-Value
Macronutrients (Intake Per Day)												
Total protein (g/1000 kcal)	33.6	31.9, 35.2	31.0	30.4, 31.6	2.6	0.010	33.2	31.7, 34.6	31.7	31.1, 32.3	1.5	0.067
Total carbohydrates (g/1000 kcal)	137.1	130.9, 143.2	144.2	142.0, 146.4	−7.1	0.038	145.8	139.6, 152.0	148.9	147.1, 150.8	−3.1	0.354
Fiber (g/1000 kcal)	14.0	13.0, 14.9	13.0	12.7, 13.3	1.0	0.055	14.2	13.3, 15.1	14.0	13.7, 14.3	0.2	0.699
Total sugars (g/1000 kcal)	52.3	46.9, 57.7	56.0	54.1, 57.9	−3.7	0.187	55.1	50.3, 59.9	60.8	58.8, 62.9	−5.7	0.041
Added sugars (g/1000 kcal)	24.9	20.6, 29.2	34.7	32.8, 36.6	−9.8	0.000	26.8	22.0, 31.6	35.0	32.9, 37.1	−8.2	0.003
Total fatty acids (g/1000 kcal)	34.2	32.2, 36.3	32.1	31.5, 32.8	2.1	0.059	32.9	30.9, 34.9	32.8	32.1, 33.4	0.1	0.938
Saturated fatty acids (g/1000 kcal)	12.9	11.7, 14.1	11.5	11.2, 11.8	1.4	0.025	12.8	11.6, 13.9	12.1	11.9, 12.4	0.7	0.322
Monounsaturated fatty acids (g/1000 kcal)	12.0	11.0, 13.0	11.1	10.8, 11.3	0.9	0.082	10.5	9.6, 11.4	11.0	10.8, 11.3	−0.5	0.252
Polyunsaturated fatty acids (g/1000 kcal)	8.0	7.3, 8.6	7.7	7.5, 7.9	0.3	0.473	7.3	6.7, 7.8	7.8	7.6, 8.1	−0.5	0.062
Trans fat (g/1000 kcal)	0.3	0.2, 0.3	0.2	0.2, 0.2	0.1	0.206	0.3	0.2, 0.3	0.3	0.3, 0.3	0.0	0.556
Omega-3 (g/1000 kcal)	0.05	0.04, 0.07	0.05	0.04, 0.05	0.00	0.487	0.04	0.03, 0.06	0.05	0.04, 0.05	0.01	0.687

^a^ Sex-specific multivariable linear regression models were used to predict macronutrient intakes according to diabetes status and adjusting for age, hypertension status, body mass index, smoking status, educational level, wealth index, rural/urban area, and geographic region. Estimates were weighted to adjust for unequal probability of sampling and to be nationally representative. CI—confidence intervals.

**Table 5:** Sex-stratified adjusted intakes of micronutrients in Mexican adults by diabetes status (*n =* 2762) ^a^.

Variables	Men						Women					
	Diabetes		No Diabetes				Diabetes		No Diabetes			
*N*	82		1060				159		1461			
	Mean	95% CI	Mean	95% CI	Diff Diab vs Non-diab	*p*-Value	Mean	95% CI	Mean	95% CI	Diff Diab vs. Non-Diab	*p*-Value
Micronutrients (Intake Per Day)												
Vitamin A (RAE) (μg/1000 kcal)	336.5	295.9, 377.0	314.6	298.2, 331.0	21.9	0.345	428.4	373.8, 483.1	404.2	386.6, 421.8	24.2	0.390
Vitamin D * (μg/1000 kcal)	2.3	1.8, 2.8	1.8	1.7, 1.9	0.5	0.065	2.2	1.8, 2.5	2.0	1.9, 2.1	0.2	0.274
Vitamin E (mg/1000 kcal)	3.5	3.1, 3.9	3.1	3.0, 3.2	0.4	0.072	3.2	3.0, 3.4	3.4	3.3, 3.5	−0.2	0.087
Folate (μg/1000 kcal)	164.4	151.9, 177.0	149.7	144.6, 154.9	14.7	0.043	179.1	163.0, 195.3	177.7	172.4, 182.9	1.4	0.854
Vitamin C (mg/1000 kcal)	91.5	74.2, 108.8	79.6	74.0, 85.3	11.9	0.209	95.6	81.7, 109.6	102.5	95.9, 109.0	−6.9	0.370
Vitamin B-6 (mg/1000 kcal)	0.9	0.9, 1.0	0.9	0.9, 0.9	0.0	0.244	1.4	0.6, 2.1	1.0	0.9, 1.1	0.4	0.321
Vitamin B-12 (μg/1000 kcal)	2.3	1.9, 2.6	2.1	2.0, 2.2	0.2	0.314	2.4	2.1, 2.7	2.2	2.1, 2.3	0.2	0.254
Calcium (mg/1000 kcal)	494.9	450.2, 539.7	424.6	413.7, 435.4	70.3	0.005	549.7	508.5, 591.0	469.5	458.0, 481.0	80.2	0.000
Magnesium (mg/1000 kcal)	207.0	191.6, 222.3	188.1	184.1, 192.1	18.9	0.022	215.3	200.2, 230.4	197.1	193.4, 200.8	18.2	0.022
Zinc (mg/1000 kcal)	5.3	5.0, 5.6	5.0	4.9, 5.0	0.3	0.037	5.5	5.3, 5.7	5.1	5.0, 5.2	0.4	0.000
Potassium (mg/1000 kcal)	1420.1	1320.8, 1519.4	1223.7	1190.0, 1257.4	196.4	0.001	1428.1	1331.5, 1524.8	1370.1	1340.9, 1399.3	58.0	0.252
Sodium (mg/1000 kcal)	1155.0	1060.5, 1249.5	1081.6	1048.7, 1114.4	73.4	0.164	1103.2	1010.3, 1196.1	1134.2	1107.3, 1161.1	−31.0	0.541

^a^ Sex-specific multivariable linear regression models were used to predict macronutrient intakes according to diabetes status and adjusting for age, hypertension status, body mass index, smoking status, educational level, wealth index, rural/urban area, and geographic region. Estimates were weighted to adjust for unequal probability of sampling and to be nationally representative. * *p*-value < 0.05, CI—confidence intervals; RAE—retinol activity equivalents.

## Supplementary Materials

The following are available online at https://www.mdpi.com/2072-6643/12/11/3464/s1, Table S1. Sex-stratified unadjusted Mexican Alternate Healthy Eating Index Score and components in Mexican adults by diabetes status. Table S2. Sex-stratified unadjusted intakes of micronutrients in Mexican adults by diabetes status. Table S3. Sex-stratified unadjusted intakes of macronutrients in Mexican adults by diabetes status.
