# Supplementary material for: Adherence to Dietary Guidelines in Adults by Diabetes Status: Results From the 2012 Mexican National Health and Nutrition Survey
Source: Nutrients. 2020 Nov 12;12(11):3464. doi: 10.3390/nu12113464 (PMC7697878; doi:10.3390/nu12113464)
Supplement: Supplementary file 1 [file nutrients-12-03464-s001.pdf]

Supplementary Material

Table S1. Sex-stratified unadjusted Mexican Alternate Healthy Eating Index Score and components in Mexican adults by diabetes status (*n*=2762)[a].

**Variables**		**Men**						**Women**					
		**Diabetes**		**Non-Diabetes**				**Diabetes**		**Non-Diabetes**			
N		**82**		**1060**				**159**		**1461**			
	Maximum Points	Mean	95% CI	Mean	95% CI	Diff diab vs non-diab	*p*-value	Mean	95% CI	Mean	95% CI	Diff diab vs non-diab	*p*-value
Total scores	100	48.0	45.5, 50.4	44.4	44.0, 45.4	3.6	0.011	46.6	45.4, 47.8	45.1	44.3, 45.8	1.5	0.034
Component scores													
Higher intake recommended													
Vegetables	9	6.4	5.4, 7.4	6.2	5.9, 6.5	0.2	0.748	5.6	5, 6.2	6.2	6, 6.4	-0.6	0.068
Whole fruit	9	4.2	3.3, 5.1	2.9	2.6, 3.1	1.3	0.005	2.7	2.2, 3.1	3.3	3.1, 3.5	-0.6	0.009
Fiber from cereals	9	2.3	1.8, 2.8	2.2	2, 2.3	0.1	0.640	1.7	1.3, 2.1	2.0	1.9, 2.1	-0.3	0.124
Nuts	5	0.3	0.1, 0.4	0.2	0.2, 0.3	0.1	0.833	0.2	0.1, 0.2	0.2	0.1, 0.2	0.0	0.641
Legumes	5	3.7	3.2, 4.1	3.5	3.3, 3.6	0.2	0.397	3.2	2.8, 3.7	3.1	3, 3.3	0.1	0.713
EPA+DHA	9	3.1	2.5, 3.8	2.9	2.7, 3.1	0.2	0.499	1.7	1.3, 2.1	2.6	2.4, 2.7	-0.9	0.000
Polyunsaturated Fats	9	5.9	5.2, 6.6	5.6	5.4, 5.8	0.3	0.505	5.3	4.7, 5.8	5.7	5.5, 5.9	-0.4	0.159
Limited intake recommended													
SSBs	9	4.3	3.2, 5.4	3.1	2.8, 3.8	1.2	0.010	6.2	5.4, 6.9	4.3	4, 4.6	1.9	0.000
Red/processed meat	9	4.3	3.8, 4.9	4.0	3.7, 4.3	0.3	0.347	5.6	5, 6.3	4.7	4.5, 5	0.9	0.010
Sodium	9	3.2	2.1, 4.3	3.2	2.8, 3.6	0.0	0.979	5.4	4.5, 6.3	4.0	3.6, 4.3	1.4	0.004
Trans Fats	9	9.0	9, 9	9.0	9, 9	0.0	0.383	9.0	9, 9	9.0	9, 9	0.0	0.677
Alcohol	9	1.34	0.43, 2.25	1.56	1.26, 1.85	-0.22	0.657	0.03	-0.03, 0.1	0.01	0.01, 0.02	0.02	0.536

aSex-specific multivariable linear regression models were used to predict dietary scores according to diabetes status. Estimates were weighted to adjust for unequal probability of sampling and to be nationally representative. CI—confidence intervals; DHA—docosahexaenoic acid; EPA— eicosapentaenoic acid; SSBs—sugar-sweetened beverages.

Table S2. Sex-stratified unadjusted intakes of micronutrients in Mexican adults by diabetes status (*n*=2762)[a].

**Variables**	**Men**						**Women**					
	**Diabetes**		**No Diabetes**				**Diabetes**		**No Diabetes**			
N	**82**		**1060**				**159**		**1461**			
	Mean	95% CI	Mean	95% CI	Diff diab vs non-diab	*p*-value	Mean	95% CI	Mean	95% CI	Diff diab vs non-diab	*p*-value
Micronutrients (intake per day)												
Vitamin A (RAE) (μg/1000kcal)	359.2	328.2, 390.3	312.7	294.7, 330.6	46.5	0.011	441.8	386.6, 497	402.0	384.6, 419.3	39.8	0.179
Vitamin D* (μg/1000kcal)	2.5	1.8, 3.1	1.7	1.6, 1.8	0.8	0.037	2.2	1.8, 2.6	1.9	1.8, 2	0.3	0.165
Vitamin E (mg/1000kcal)	3.5	3, 3.9	3.1	3, 3.1	0.4	0.093	3.3	3.1, 3.5	3.4	3.3, 3.5	-0.1	0.159
Folate (μg/1000kcal)	169.4	157.5, 181.3	147.9	142.7, 153	21.5	0.001	185.9	169.7, 202	177.7	171.9, 183.5	8.2	0.343

Vitamin C (mg/1000kcal)	94.2	76.6, 111.9	78.2	72.5, 83.9	16.0	0.094	93.3	80.9, 105.8	101.6	95.3, 107.9	-8.3	0.246
Vitamin B-6 (mg/1000kcal)	1.0	0.9, 1	0.9	0.9, 0.9	0.1	0.012	1.3	0.7, 1.9	1.0	0.9, 1.1	0.3	0.275
Vitamin B-12 (μg/1000kcal)	2.6	2.2, 3	2.1	1.9, 2.2	0.5	0.009	2.5	2.2, 2.7	2.2	2.1, 2.3	0.3	0.096
Calcium (mg/1000kcal)	510.1	469.9, 550.4	422.5	412, 433	87.6	0.000	554.6	511.2, 598	471.1	460.2, 482	83.5	0.000
Magnesium (mg/1000kcal)	208.1	193.1, 223	186.8	182.7, 190.9	21.3	0.008	226.9	213.1, 240.8	197.9	194.2, 201.6	29.0	0.000
Zinc (mg/1000kcal)	5.4	5.1, 5.6	4.9	4.9, 5	0.5	0.003	5.5	5.3, 5.7	5.1	5, 5.2	0.4	0.001
Potasium (mg/1000kcal)	1487.1	1372.8, 1601.4	1206.7	1173.2, 1240.2	280.4	0.000	1472.5	1373.8, 1571.2	1371.6	1341.3, 1401.9	100.9	0.049
Sodium (mg/1000kcal)	1144.0	1057.4, 1230.6	1077.1	1044.1, 1110.2	66.9	0.157	1116.7	1019.6, 1213.8	1143.2	1115.6, 1170.8	-26.5	0.617

aSex-specific multivariable linear regression models were used to predict macronutrient intakes according to diabetes status. Estimates were weighted to adjust for unequal probability of sampling and to be nationally representative. * p-value < 0.05. CI—confidence intervals; RAE—retinol activity equivalents.

Table S3. Sex-stratified unadjusted intakes of macronutrients in Mexican adults by diabetes status (n=2762)[a].

**Variables**	**Men**						Women					
	**Diabetes**		**Non-Diabetes**				**Diabetes**		**Non-Diabetes**			
N	**82**		**1060**				**159**		**1461**			
	Mean	95% CI	Mean	95% CI	Diff diab vs non-diab	p-value	Mean	95% CI	Mean	95% CI	Diff diab vs non-diab	p-value
	Macronutrients (intake per day)											
Total protein (g/1000kcal)	34.4	33, 35.8	30.7	30.1, 31.4	3.7	0.000	33.7	32.3, 35.1	31.9	31.4, 32.4	1.8	0.019
Total carbohydrates (g/1000kcal)	135.7	129.9, 141.5	143.6	141.3, 145.9	-7.9	0.012	147.4	141.4, 153.3	149.2	147.5, 150.8	-1.8	0.564
Fiber (g/1000 kcal)	13.6	12.6, 14.6	12.9	12.6, 13.3	0.7	0.190	14.7	13.8, 15.6	14.2	13.9, 14.5	0.5	0.294
Total sugars (g/1000kcal)	53.5	48.4, 58.7	55.5	53.6, 57.3	-2.0	0.490	53.2	48.7, 57.7	60.1	58.3, 61.9	-6.9	0.008
Added sugars (g/1000kcal)	24.2	20.8, 27.7	34.7	33, 36.5	-10.5	0.000	23.7	19.4, 28.1	34.1	32.1, 36	-10.4	0.000
Total fatty acids (g/1000kcal)	35.2	32.9, 37.6	31.8	31.1, 32.5	3.4	0.006	32.7	30.9, 34.4	32.8	32.3, 33.3	-0.1	0.897
Saturated fatty acids (g/1000kcal)	13.7	12.2, 15.2	11.4	11.1, 11.7	2.3	0.004	12.5	11.5, 13.6	12.1	11.8, 12.3	0.4	0.403
Monounsaturated fatty acids (g/1000kcal)	12.3	11.1, 13.6	11.0	10.7, 11.2	1.3	0.040	10.4	9.6, 11.1	11.0	10.8, 11.2	-0.6	0.105
Polyunsaturated fatty acids (g/1000kcal)	7.9	7.1, 8.6	7.6	7.4, 7.8	0.3	0.553	7.5	6.9, 8.1	7.9	7.7, 8.1	-0.4	0.199
Transfat (g/1000kcal)	0.3	0.2, 0.3	0.2	0.2, 0.2	0.1	0.191	0.3	0.2, 0.3	0.3	0.2, 0.3	0.0	0.789
Omega-3 (g/1000kcal)	0.05	0.04, 0.06	0.05	0.04, 0.06	0.00	0.424	0.04	0.03, 0.06	0.05	0.04, 0.06	-0.01	0.610

aSex-specific multivariable linear regression models were used to predict macronutrient intakes according to diabetes status. Estimates were weighted to adjust for unequal probability of sampling and to be nationally representative. CI—confidence intervals.
